# RIM-Binding Proteins Are Required for Normal Sound-Encoding at Afferent Inner Hair Cell Synapses

**DOI:** 10.3389/fnmol.2021.651935

**Published:** 2021-03-23

**Authors:** Stefanie Krinner, Friederike Predoehl, Dinah Burfeind, Christian Vogl, Tobias Moser

**Affiliations:** ^1^Institute for Auditory Neuroscience and InnerEarLab, University Medical Center Göttingen, Göttingen, Germany; ^2^Collaborative Research Center 1286, University of Göttingen, Göttingen, Germany; ^3^Auditory Neuroscience Group, Max Planck Institute of Experimental Medicine, Göttingen, Germany; ^4^Presynaptogenesis and Intracellular Transport in Hair Cells Group, Institute for Auditory Neuroscience and InnerEarLab, University Medical Center Göttingen, Göttingen, Germany; ^5^Multiscale Bioimaging Cluster of Excellence, University of Göttingen, Göttingen, Germany

**Keywords:** RIM-BP, calcium, exocytosis, active zone (AZ), cochlea, hearing, ribbon synapse

## Abstract

The afferent synapses between inner hair cells (IHC) and spiral ganglion neurons are specialized to faithfully encode sound with sub-millisecond precision over prolonged periods of time. Here, we studied the role of Rab3 interacting molecule-binding proteins (RIM-BP) 1 and 2 – multidomain proteins of the active zone known to directly interact with RIMs, Bassoon and Ca_*V*_1.3 – in IHC presynaptic function and hearing. Recordings of auditory brainstem responses and otoacoustic emissions revealed that genetic disruption of RIM-BPs 1 and 2 in mice (*RIM-BP1/2^–/–^*) causes a synaptopathic hearing impairment exceeding that found in mice lacking RIM-BP2 (*RIM-BP2^–/–^*). Patch-clamp recordings from *RIM-BP1/2^–/–^* IHCs indicated a subtle impairment of exocytosis from the readily releasable pool of synaptic vesicles that had not been observed in *RIM-BP2^–/–^* IHCs. In contrast, the reduction of Ca^2+^-influx and sustained exocytosis was similar to that in RIMBP2^–/–^ IHCs. We conclude that both RIM-BPs are required for normal sound encoding at the IHC synapse, whereby RIM-BP2 seems to take the leading role.

## Introduction

The ribbon-type active zones (AZ) of inner hair cells (IHCs) are molecularly specialized to ensure temporally precise encoding of incoming sound stimuli into high-frequency firing of postsynaptic spiral ganglion neurons (SGNs). In mature mouse IHCs, neurotransmitter release from synaptic vesicles (SV) is controlled by the tight spatial coupling of Ca_*V*_1.3 voltage-gated L-type Ca^2+^-channels and the SV release machinery (Brandt et al., 2005; Wong et al., 2014; Pangrsic et al., 2015). A small, defined SV pool with fast release kinetics, referred to as the readily releasable pool (RRP), is much less sensitive to the slow-binding Ca^2+^-buffer EGTA than to the fast-binding Ca^2+^-buffer BAPTA, emphasizing the Ca^2+^-nanodomain-like control of SV release (Moser and Beutner, 2000; Brandt et al., 2005; Pangrsic et al., 2015). There is also evidence for Ca^2+^ nanodomain-like coupling in hair cells of other species, such as in frog auditory hair cells (Graydon et al., 2011), in rat IHCs (Goutman and Glowatzki, 2007) and IHCs of the low frequency apical cochlea of the gerbil, while a looser Ca^2+^ microdomain-like coupling was reported for the high-frequency basal IHCs (Johnson et al., 2017) and immature mouse IHCs (Wong et al., 2014).

Aside from the sophisticated Ca^2+^-channel complex (reviewed in Pangrsic et al., 2018), the release of SVs in auditory IHCs also requires a finely coordinated, complex presynaptic protein network including bassoon, piccolo, Rab3-interacting molecule (RIM) 2α/2ß/3γ and RIM-binding protein (RIM-BP), organizing the precise AZ topography of Ca^2+^-channels and SV release sites (reviewed in Moser et al., 2019). RIM-BPs seem to take a central role in a large presynaptic multiprotein-complex involving voltage-gated Ca^2+^-channels, RIMs and bassoon (Hibino et al., 2002; Kaeser et al., 2011; Davydova et al., 2014; Acuna et al., 2015; Ortner et al., 2020). Specifically, the RIM-BPs’ two C-terminal SH3 domains interact with proline-rich motifs in the C-terminus of the Ca_*V*_α_1D_ subunit and RIMs (Wang et al., 2000; Hibino et al., 2002; Kaeser et al., 2011; Ortner et al., 2020; Petzoldt et al., 2020), while the N-terminal SH3 domain interacts with the proline-rich motif of bassoon (Davydova et al., 2014). Morphological and functional studies from mouse IHCs suggest that these interactions are likely also applicable to IHC ribbon-type AZs. On the one hand, super-resolution microscopy studies on immunolabeled IHCs showed a specific stripe-like arrangement of the above mentioned interaction partners bassoon (Wong et al., 2014; Neef et al., 2018), RIM2 (Jung et al., 2015), RIM-BP2 (Krinner et al., 2017), and Ca_*V*_1.3 Ca^2+^-channels (Frank et al., 2010; Neef et al., 2018) at IHC AZs. On the other hand, the individual genetic deletion of bassoon (Khimich et al., 2005; Frank et al., 2010; Neef et al., 2018), RIM2α and -ß (Jung et al., 2015) and RIM-BP2 (Krinner et al., 2017) significantly reduced the number of IHC presynaptic Ca_*V*_1.3 Ca^2+^-channels. While these data show that all three proteins are important for Ca^2+^-channel clustering, none of the mentioned mutants exhibit a complete loss of synaptic Ca_*V*_1.3 Ca^2+^-channels or exocytosis, suggesting partially overlapping and compensatory function between these presynaptic AZ proteins to ensure normal presynaptic IHC function. In the retina, for example, RIMs were found to be upregulated upon loss of RIM-BP1/2 (Luo et al., 2017), while nonetheless a significant reduction of synaptic Ca^2+^-channels was observed at rod bipolar cell ribbon-type AZs (Luo et al., 2017). The spectrum of effects of RIM-BP deletion on presynaptic function ranges across synapses. In *Drosophila melanogaster* neuromuscular junctions (NMJ), genetic disruption of DRPB (RIM-BP orthologue) causes a severe impairment of Ca^2+^-channel clustering (Liu et al., 2011; Müller et al., 2015), while at conventional synapses of the mammalian CNS or *Caenorhabditis elegans* synapses, the number of P/Q- or N-type Ca^2+^-channels was not affected by the loss of RIM-BPs (Acuna et al., 2015; Grauel et al., 2016; Kushibiki et al., 2019). Genetic disruption of DRPB in *D. melanogaster* NMJ further affected the structural AZ integrity and functional coupling between Ca^2+^-channels and SVs, resulting in a drastically reduced SV release probability (Liu et al., 2011; Müller et al., 2015). Such looser SV-Ca^2+^-channel coupling upon RIM-BP-disruption was also observed in conventional CNS synapses and retinal ribbon synapses (Acuna et al., 2015; Grauel et al., 2016; Luo et al., 2017), whereas the tight nanodomain-like coupling remained unaltered in IHC ribbon synapses at least after recovery of the RRP from depletion (Krinner et al., 2017).

RIM-BPs also contribute to the efficient replenishment of readily releasable SVs. In *D. melanogaster* NMJ, the N-terminal deletion of DRBP lead to impaired SV recruitment to release sites, mediated via DRBP – Bruchpilot (BRP)/ELKS/CAST interaction (Petzoldt et al., 2020). Impaired SV replenishment was also reported for calyx of Held (Acuna et al., 2015) and ribbon synapses (Krinner et al., 2017; Luo et al., 2017). For IHC AZs, this has been suggested to reflect a role of RIM-BP2 in registering new-coming SVs in nanoscale proximity of Ca^2+^ channels (Krinner et al., 2017). Recently, a role of RIM-BP in SV priming via interaction with Munc13-1 has been suggested (Brockmann et al., 2020). As priming of SVs in IHCs seems to operate without Munc13 and CAPS priming proteins (Vogl et al., 2015), other protein interactions such as the one with CAST/ELKS (Petzoldt et al., 2020) remain to be studied for a potential involvement in SV replenishment. In support of the relevance of this interaction at mammalian synapses, we note that RIM-BP expression levels are linked to CAST/ELKS abundance. In hippocampal neurons, ELKS and RIM deletion caused reduced protein levels of RIM-BP2 (Wang et al., 2016), while ELKS was found to be upregulated in RIM-BP1/2 deficient retinae (Luo et al., 2017).

*In vivo* experiments on RIM-BP2 knockout mice (*RIM-BP2^–^*^/^*^–^*) revealed a mild synaptopathic hearing impairment (Krinner et al., 2017). Likewise, the genetic deletion of the above mentioned AZ proteins and RIM-BP interaction partners bassoon (Khimich et al., 2005) and RIM2α (Jung et al., 2015) caused a significant, yet limited, elevation of hearing thresholds. Hence, the auditory system phenotype suggests overlapping and compensatory function of these AZ proteins, which is in agreement with the cell physiology. Yet, the consequences of RIM-BP2 loss-of-function might have been attenuated by the presence of other RIM-BP variants, such as RIM-BP1 or -3. Hence, we tested the presence and potential role of RIM-BP1 in cochlear function by comparing RIM-BP1/2 double-knockout mouse line (*RIM-BP1/2^–^*^/^*^–^*) (Grauel et al., 2016), to the previously studied *RIM-BP2^–^*^/^*^–^* mice (Krinner et al., 2017). We employed expression analysis, electrophysiology and systems physiology and, indeed, found a synaptopathic hearing impairment in *RIM-BP1/2^–^*^/^*^–^* mice that exceeds that of *RIM-BP2^–^*^/^*^–^* mice. In IHC physiology, additional deletion of RIM-BP1 caused a subtle impairment of RRP exocytosis not found in *RIM-BP2^–^*^/^*^–^* IHCs, suggesting that both RIM-BPs (-1 and -2) are required for normal hearing and sound encoding at the IHC ribbon synapse.

## Materials and Methods

### Animals

*RIM-BP1/2* double-knockout mice (*RIM-BP1/2^–/–^*) (Grauel et al., 2016) and *C57BL*/*6* mice of either sex were used for experiments. Previously published data from *RIM-BP2* knockout mice (*RIM-BP2^–/–^*) and their *RIM-BP2* wild-type littermates (*RIM-BP2^+/+^*) were used for comparison as indicated (Krinner et al., 2017). Electrophysiology, RNAscope and immunohistochemistry experiments were performed in 2–3 week-old mice (i.e. after hearing-onset). Systems physiology was carried out in 8–10 week-old mice. All experiments complied with national animal care guidelines and were approved by the University of Göttingen board for animal welfare and the animal welfare office of the state of Lower Saxony.

### Immunohistochemistry and Confocal Microscopy

Immunohistochemistry was carried out essentially as described in Khimich et al. (2005). If not stated differently, all steps were carried out at room temperature. Apical turns of 2–3 week-old *RIM-BP1/2^–/–^* and *C57BL/6* mouse organs of Corti were dissected in phosphate buffered saline (PBS) and fixed with 4% formaldehyde (FA) in PBS on ice for 10 min. After fixation, the organs of Corti were washed 3 × 10 min in PBS and incubated in goat serum dilution buffer (GSDB: 16.7% normal goat serum, 450 mM NaCl, 0.3% Triton X-100, 20 mM phosphate buffer, pH 7.4) in a wet chamber. Thereafter, primary antibodies were applied overnight in a wet chamber at 4°C. After washing the organs of Corti 3 × 10 min (wash buffer: 450 mM NaCl, 20 mM phosphate buffer, 0.3% Triton X-100), they were incubated with secondary antibodies for 1 h in a wet and light-protected chamber. Finally, organs of Corti were washed 3 × 10 min in wash buffer, 10 min in 5 mM phosphate buffer and mounted on glass microscopy slides with a drop of fluorescence mounting medium (Mowiol). The following antibodies have been used: mouse-IgG1-anti-CtBP2 (also recognizing the ribbon protein RIBEYE, BD Biosciences, 1:200), guinea pig-anti-synapsin1/2 (Synaptic Systems, 1:500), guinea pig-anti-bassoon (Synaptic Systems, 1:500), rabbit-anti-RIM-BP1 (Synaptic Systems, 1:200), rabbit-anti-calretinin (Swant, 1:1,000) and AlexaFluor647 goat-anti-rabbit (Invitrogen, 1:200), STAR470spx-, STAR580-, and Star635P-labeled goat-anti-rabbit, goat-anti-mouse and goat-anti-guinea pig antibodies (Abberior, 1:200). All antibodies were diluted in GSDB. Mutant and wild-type samples were treated in parallel. Confocal images were acquired with 488, 561, and 640 nm excitation lasers, and a 100× oil immersion objective (1.4 NA, Olympus) using an Abberior Expert Line STED microscope (Abberior Instruments, Göttingen, Germany). Images were acquired, using identical laser power and microscope settings. Images were analyzed using ImageJ and assembled in Adobe Illustrator software.

### RNAscope

Cochleae from 2-week-old *C57BL/6* mice were fixed for 2 h on ice in 4% FA in DEPC-treated PBS. Apical turns of the organs of Corti were dissected in DEPC-treated PBS. Free floating organs of Corti were dehydrated with EtOH (1× 50% 5 min, 1× 70% 5 min, 2× 100% 5 min) and left to dry for a few minutes. The RNAscope assay was performed in accordance with the manufacture’s protocol (RNAscope^®^ Multiplex Reagent Kit User Manual, 320293-USM, ACDbio). Protease III was applied and incubated for 30 min at 40°C. Afterward, samples were washed twice for 5 min in water (DEPC-treated, autoclaved). The probes (RIM-BP1-C1, Otoferlin-C2, ACDbio) were applied (1:50 dilution, 50–70 μl) and incubated for 2 h at 40°C. Tissue was washed twice with wash buffer for 2 min. Finally, amplification steps were performed at 40°C: Amp 1-FL 30 min, Amp 2-FL 15 min, Amp 3-FL 30 min, Amp 4-FL (Variant A: RIM-BP1-C1 with Alexa 488 fluorophore, Otoferlin-C2 with ATTO 550 fluorophore) 15 min. Samples were washed in wash buffer for 2 min and twice in PBS for 2 min. Then, immunohistochemistry and confocal imaging were performed as described above. The RIM-BP1 RNAscope probe was custom made by ACDbio targeting the region 5565–5927 of RIM-BP1 (NM_172449.2).

### RT-PCR and Single-Cell Nested RT-PCR

*C57BL/6* mice at the age of postnatal days (p) 14 through 16 were used to determine the general expression of RIM-BPs in the organ of Corti and in single IHCs. For RT-PCR of organs of Corti we isolated total RNA from preparations of the organ of Corti and brain (control) using TRIzolReagent (Invitrogen). Reverse transcription was performed with SuperScriptII RT according to the manufacturer’s instructions using oligo (dT) primers. Sequences of RIM-BP-specific primers are listed in Table 1 and only the first PCR was run with less than 30 cycles. For single-cell PCR, individual IHCs from the apical coils of freshly dissected organs of Corti were harvested after cleaning off supporting cells at a high bath perfusion rate (3 ml/min). Ten IHCs per trial were collected to check the expression of RIM-BP1 and -2. Each individual IHC was aspirated into a glass pipette and the pipette content was transferred into first strand cDNA synthesis mix containing after the dilution: 50 mM Tris–HCl, pH 8.3, 75 mM KCl, 5 mM MgCl_2_, 5 mM DTT, 100 units of SuperScript II Reverse Transcriptase (Invitrogen, Carlsbad, CA, United States) and 40 units RNaseOUT Ribonuclease inhibitor (Invitrogen). Reverse transcription was performed with oligo (dT) primers according to the manufacturer’s instructions. Each cDNA mix was used as a template for two subsequent PCR reactions with nested primers specific for RIM-BP1 or RIM-BP2 cDNA. Instead of cDNA, distilled water was used in the negative control reaction. Single-cell nested RT-PCR was performed three times.

**TABLE 1:** Primers for nested RT-PCR.

	1st	Nested
RIM-BP1 forward	TGGGCAAGGAAGGTC CCCAGT	GATCGCCCTGCGCA ACCAGC
RIM-BP1 reverse	TCCTCCACCAGGCG GGCATT	GCAGAGCTCAGACTC CAGCTGC
RIM-BP2 forward	CCTGGCCTTCCTCAATG CCAAGC	GAGCATGAAGGTGCTG TGCAGCT
RIM-BP2 reverse	GTTGTAACTGTAGCGGG CCACACAC	CCGGTCATTGTCCATC TCGGACT

### Patch-Clamp Recordings From Inner Hair Cells and Analysis

Perforated patch-clamp recordings were essentially carried out as described in Moser and Beutner (2000). Apical turns of 2–3 week-old *RIM-BP1/2^–/–^* mouse organs of Corti were freshly dissected and whole-cell Ca^2+^-current and exocytosis from IHCs were recorded at room temperature (22–24°C). The extracellular patch-clamp solution contained (in mM): 110 NaCl, 35 TEA-Cl, 2.8 KCl, 2 CaCl_2_, 1 MgCl_2_, 10 NaOH-HEPES, 11.3 D-glucose, pH 7.3. The internal pipette solution contained (in mM): 130 Cs-gluconate, 10 TEA-Cl, 10 4-AP, 10 HEPES, 1 MgCl_2_, amphotericin B (300 μg/ml), pH 7.2. The patch-clamp used an EPC-10 amplifier and Patchmaster software (HEKA Elektronik, Lambrecht, Germany). Voltages were corrected for liquid junction potentials (14 mV) and currents were leak-corrected using a p/10 protocol. For analysis Igor Pro software (Wavemetrics, Lake Oswego) was used. For membrane capacitance (C_*m*_) measurements, IHCs were stimulated by depolarizations to −14 mV with intervals of 60–120 s to allow for recovery of IHC exocytosis. For current–voltage relationships (IVs) measurements, currents were evoked by 10 ms step depolarizations to various potentials from −100 to +30 mV in 5 mV increments. IVs were calculated from the currents during the last 8 ms of the step depolarization. From these, fractional activation curves were calculated by calculating the Ca^2+^ conductance from the Ca^2+^-current (*I*_*Ca*_) as $G\,\left(V\right)=\frac{I_{\text{Ca}}}{\left(V-V_{\text{rev}}\right)}$, with *V* the command potential and *V*_*rev*_ the reversal potential of the current obtained from the *x*-axis crossing of an extrapolating line fit to the currents from 6 to 26 mV. After normalizing these traces to the maximum conductance in the range of −20 to 10 mV, they were fit with a Boltzmann equation $G_{n}\,\left(V\right)=\frac{G_{\text{n},\max}}{1+e^{\frac{V_{\text{half}}-V}{k}}}$ with *G*_*n,m*__*ax*_, the maximum conductance, *V* the command potential, *V*_*half*_ the voltage of half-maximal activation, and *k* the slope factor. We measured C_*m*_ changes (ΔC_*m*_) using the Lindau-Neher technique (Lindau and Neher, 1988) as previously described (Moser and Beutner, 2000). Briefly, the exocytic ΔC_*m*_ was quantified as the difference of the averaged C_*m*_ 400 ms before and after the depolarization. To avoid impact of C_*m*_-transients related to conductance or gating of ion channels on ΔC_*m*_ estimation (Moser and Beutner, 2000; Neef et al., 2007) we skipped the first 100 ms of post-depolarization C_*m*_ for estimating the average. Mean ΔC_*m*_ and Ca^2+^-current estimates present grand averages calculated from the mean estimates of individual IHCs, where each depolarization was repeated 2-3 times. This avoided dominance of IHCs contributing more sweeps.

### Systems Physiology: Auditory Brainstem Responses and Distortion Product Otoacoustic Emissions

Auditory brainstem responses (ABR) and distortion product otoacoustic emissions (DPOAE) were performed as described in Jing et al. (2013) and Strenzke et al. (2016). In this study, 8–10 week-old *RIM-BP1/2^–/–^* mice were anesthetized with ketamine (125 mg/kg) and xylazine (2.5 mg/kg) i.p. and the core body temperature was maintained constant at 37°C using a heat blanket (Hugo Sachs Elektronik–Harvard Apparatus). A TDT II System run by BioSig software (Tucker Davis Technologies) was used for stimulus generation, presentation, and data acquisition. With a JBL 2402 speaker, tone bursts (4/6/8/12/16/24/32 kHz, 10 ms plateau, 1 ms cos2 rise/fall) or clicks of 0.03 ms were presented ipsilaterally in the free field at 40 Hz (tone bursts) or 20 and 100 Hz (clicks). The difference potential between vertex and mastoid subdermal needles was amplified 50,000 times, filtered (400–4,000 Hz) and sampled at a rate of 50 kHz for 20 ms, 1,300 times, to obtain two mean ABR traces for each sound intensity. Hearing thresholds were determined with 10 dB precision as the lowest stimulus intensity that evoked a reproducible response waveform in both traces by visual inspection by two independent observers. For DPOAE, continuous primary tones [frequency f2 = 1.2^∗^f1, intensity l2 = l1 − 10 decibel (dB) Sound pressure levels (SPL)] were delivered through the MF1 speaker system (Tucker Davis Technologies) and a custom-made probe containing a MKE-2 microphone (Sennheiser). The microphone signal was amplified (DMX 6Fire, Terratec) and the DPOAE amplitude at 2^∗^f2-f1 was analyzed by fast Fourier transformation using custom-written Matlab software (Mathworks). SPL are provided in dB SPL root mean square (RMS) (tonal stimuli) or dB SPL peak equivalent (clicks).

### Statistical Data Analysis

For statistical data analysis, Igor Pro software (Wavemetrics) and Origin software (Originlab) were used. Normality of distribution was tested with the Jarque-Bera test and variances were compared with the *F*-test. Unpaired, two-tailed Wilcoxon rank test (Mann–Whitney *U* test) was used to compare non-normal data or data with unequal variances, else Student’s *t*-test was employed. For patch-clamp capacitance, ABR and DPOAE data, a one-way ANOVA was used for multiple comparisons followed by *post hoc* Tukey’s test. Data are presented as mean ± SEM. Data from RIM-BP1/2 double-knockout mice (*RIM-BP1/2^–/–^*) are presented in magenta, data from RIM-BP2 knockout mice (*RIM-BP2^–/–^*) are presented in green, and data from their wildtype littermates (*RIM-BP2^+/+^*) are presented in black.

## Results

### RIM-BP1 Is Expressed in the Mouse Organ of Corti

Previous studies showed a localization of RIM-BP2 to ribbon-type AZs of IHCs and presynaptic terminals of efferent lateral olivocochlear neurons (Krinner et al., 2017; Ortner et al., 2020). Such RIM-BP2 immunofluorescence was abolished in RIM-BP2-deficient IHCs demonstrating a specific labeling by the RIM-BP2 antibody. Here, we tested the hypothesis of a comparable expression pattern of RIM-BP1 in the mouse organ of Corti. For that we performed immunolabeling and confocal microscopy of mouse IHCs with two different triple antibody stainings for RIM-BP1, CtBP2/RIBEYE (marking the presynaptic ribbon), and either the presynaptic density marker bassoon (Supplementary Figure 1A) or synapsin 1/2 (Supplementary Figure 1B), marking the conventional presynaptic terminals of efferent lateral olivocochlear neurons (Safieddine and Wenthold, 1999). We found colocalizing immunofluorescence of RIM-BP1, CtBP2/RIBEYE and bassoon (Supplementary Figure 1A) as well as of RIM-BP1 with synapsin1/2 (Supplementary Figure 1B). Labeling for RIM-BP1, however, was also present in the RIM-BP1/2-deficient IHCs, questioning the antibody specificity. In an attempt to further clarify this issue, we investigated the expression of RIM-BP1 in IHCs on the mRNA level with two different approaches. We performed (i) the RNAscope mRNA detection assay using fluorescent RNA probes with consecutive immunohistochemistry (Supplementary Figure 2; Salehi et al., 2018) and (ii) nested RT-PCR from 2-week-old *C57Bl/6* mouse organs of Corti and individual IHCs (Supplementary Figure 3). In the nested RT-PCR, both RIM-BP1 and -2 were detected in brain tissue and the organ of Corti (Supplementary Figures 3A,B). However, while RIM-BP2 mRNA was detected in individual IHCs (Supplementary Figure 3B), RIM-BP1 was not (Supplementary Figure 3A). This finding is consistent with our results from the RNAscope assay using fluorescent RNA probes targeting RIM-BP1 (Supplementary Figure 2D) and Otoferlin (Supplementary Figure 2C) as a positive control and consecutive immunohistochemistry in which we used calretinin labelling (Supplementary Figure 2B) to visualize IHCs. While the Otoferlin RNA probe showed a clear signal around all IHC nuclei, the RIM-BP1 RNA probe resulted in sparse if any signal (Supplementary Figure 2A, merge image).

### RIM-BP2 Dominates the Function of RIM-BPs at the IHC Ribbon Synapse

**FIGURE 1:**
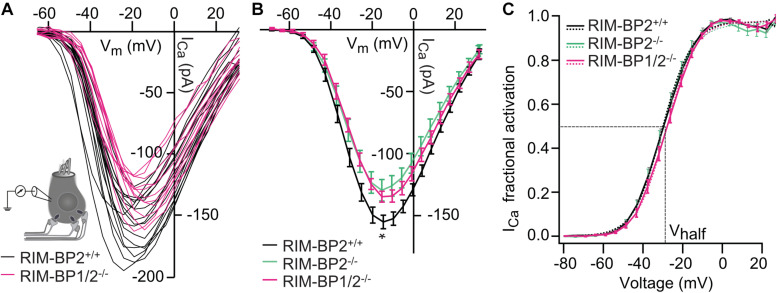
Additional RIM-BP1 disruption does not alter voltage-dependent Ca^2+^-influx. **(A)** Schematic drawing of an IHC during perforated patch-clamp experiment and raw traces of Ca^2+^-current-voltage relationships (IVs) of RIM-BP1/2-deficient (*RIM-BP1/2^–/–^*, magenta) and control (*RIM-BP2^+/+^*, black) IHCs. IVs were calculated from the last 8 ms of currents evoked by step depolarizations to various potentials. **(B)** Mean ± SEM from IVs of RIM-BP1/2-deficient (*RIM-BP1/2^–/–^*, *n* = 15, magenta), RIM-BP2-deficient (*RIM-BP2^–/–^*, *n* = 16, green) and control (*RIM-BP2^+/+^*, *n* = 17, black) IHCs in panel **(A)**. Compared to control IHCs, Ca^2+^-current amplitudes were significantly reduced in RIM-BP1/2-deficient IHCs (*p* = 0.02). However, no significant difference was found between RIM-BP1/2-deficient and RIM-BP2-deficient IHCs. **(C)** Fractional activation curves of the whole-cell Ca^2+^-current: A Boltzmann function was fit to the normalized conductance curve **(C)** calculated from the IVs **(A)**. Average fit data are displayed for all three genotypes (dashed traces*: RIM-BP1/2^–/–^*, *n* = 15, magenta, *RIM-BP2^–/–^, n* = 16, green and *RIM-BP2^+/+^*, *n* = 17, black). Dashed vertical line indicates V_*half*_, reporting the voltage of half-maximal activation of the whole-cell Ca^2+^-current. **(A–C)** Mean ± SEM and statistical *p*-values are displayed in Table 2. Data information: Data **(B,C)** represent IHC grand averages, mean ± SEM; Data **(A)** represent raw data from individual IHCs. Significance level: n.s. *p* ≥ 0.05, *p < 0.05; n = number of IHCs; age of mice: p14-p16. Data of RIM-BP2-deficient (*RIM-BP2^–/–^*) and control (*RIM-BP2^+/+^*) IHCs were adapted from Krinner et al. (2017).

**FIGURE 2:**
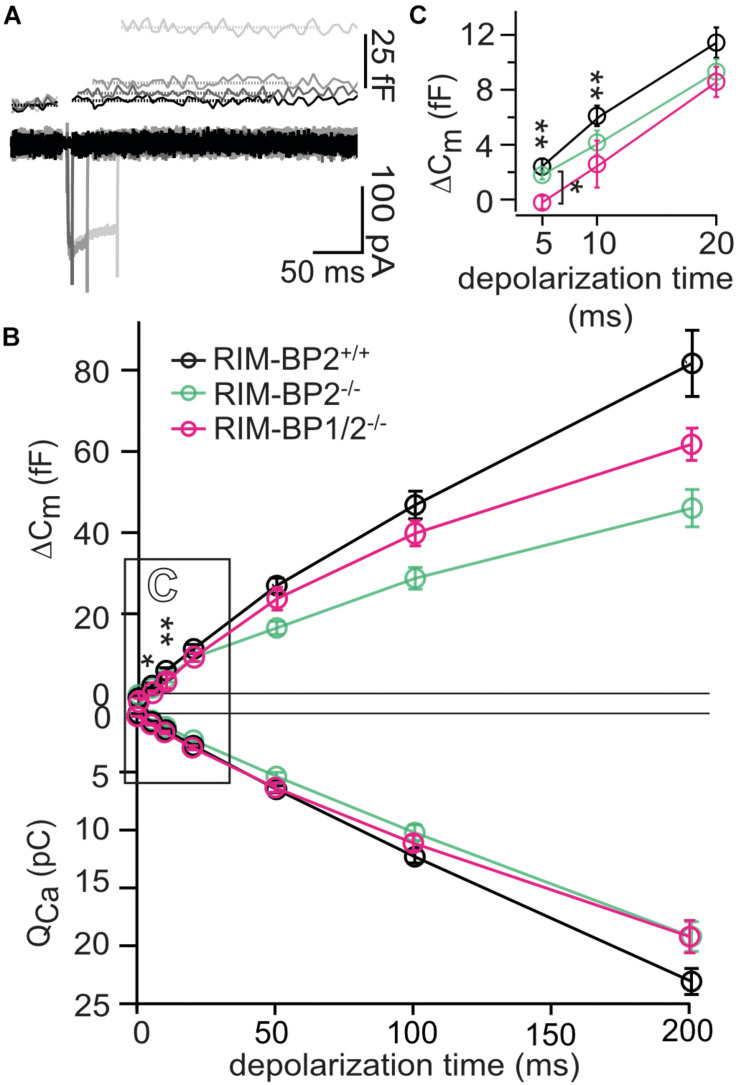
RIM-BP1 promotes exocytosis of the RRP. **(A)** Representative C_*m*_ traces and their corresponding Ca^2+^-currents (I_*Ca*_) from an individual RIM-BP1/2-deficient IHC in response to various depolarization durations to −14 mV (indicated by different gray values) from a perforated patch-clamp recording. The C_*m*_ data of the first 100 ms after the depolarization containing a non-exocytic C_*m*_ transient have been removed as they are discarded for the analysis of the exocytic C_*m*_ change (ΔC_*m*_). The horizontal lines represent the average C_*m*_ before and after the depolarization. Scale bar: 50 ms, 25 fF (C_*m*_), 100 pA (I_*Ca*_). **(B)** Relationship of ΔC_*m*_ (top) and the corresponding whole-cell Ca^2+^-current integrals (Q_*Ca*_, bottom) of RIM-BP1/2-deficient (*RIM-BP1/2^–/–^*, *n* = 8, magenta), RIM-BP2-deficient (*RIM-BP2^–/–^*, *n* = 10, green) and control (*RIM-BP2^+/+^*, *n* = 12, black) IHCs for various depolarization durations to −14 mV. **(C)** Responses to short depolarizations [black box in panel **(B)**] are magnified for better display. For various depolarization durations, exocytic ΔC_*m*_ of RIM-BP1/2-deficient IHCs differed significantly from control IHCs (indicated by asterisk on top of traces, 5 ms: *p* = 0.003, 10 ms: *p* = 0.04). Compared to RIM-BP2-deficient IHCs, a difference in exocytic ΔC_*m*_ of RIM-BP1/2-deficient IHCs was detected for 5 ms IHC depolarization duration (indicated by asterisk and bracket, *p* = 0.03). **(B,C)** Data information: Data represent IHC grand averages, mean ± SEM; one-way ANOVA and for multiple comparisons, *post-hoc* Tukey’s test, *p*-values are summarized in Table 3. Significance levels: n.s. *p* ≥ 0.05, *p < 0.05, **p < 0.01; *n* = number of IHCs; age of mice: p14-p16. Data of RIM-BP2-deficient (*RIM-BP2^–/–^*, *n* = 10, green) and control (*RIM-BP2^+/+^*, n = 12, black) IHCs were adapted from Krinner et al. (2017).

Next, we addressed the question, whether additional genetic disruption of RIM-BP1 in mice aggravates the deficit in presynaptic function of sensory IHCs beyond that found for RIM-BP2 single-knockout mice (*RIM-BP2^–/–^*). Specifically, using constitutive RIM-BP1/2 double-knockout mice (*RIM-BP1/2^–/–^*), we aimed to test whether RIM-BP1, just like RIM-BP2 (Krinner et al., 2017), promotes the synaptic Ca_*V*_1.3 Ca^2+^-channel abundance and/or whether RIM-BP1 might directly regulate the exocytosis machinery. To address these points, we performed perforated-patch whole-cell recordings from IHCs of *RIM-BP1/2^–/–^* mice to characterize IHC voltage-gated Ca^2+^-influx (Figure 1) and exocytosis (Figure 2). We compared the whole-cell Ca^2+^-current amplitude evoked by step depolarizations to various potentials of IHCs from mice lacking both RIM-BP1 and 2 (*RIM-BP1/2^–/–^*) to IHCs from control mice (*RIM-BP2^+/+^*) (Figure 1A), which was significantly reduced (*p* = 0.02; Wilcoxon rank test) (Figure 1B and Table 2). However, no statistically significant difference was found between the IHCs of *RIM-BP1/2^–/–^* mice and *RIM-BP2^–/–^* mice (data from Krinner et al., 2017). Further, consistent with the findings in RIM-BP2-deficient IHCs (Krinner et al., 2017), no difference in the voltage-dependence of Ca^2+^-channel activation was observed in RIM-BP1/2-deficient IHCs (Figure 1C and Table 2). The integral of the voltage-gated Ca^2+^-current (Q_*Ca*_), obtained for step depolarizations of varying length, showed no significant reduction for RIM-BP1/2-deficient IHCs compared to control IHCs (*RIM-BP2^+/+^*) and was comparable to RIM-BP2-deficient IHCs (Figure 2B). Hence, while RIM-BP2 is a positive regulator of synaptic Ca_*V*_1.3 Ca^2+^-channel abundance (Krinner et al., 2017) and stabilizes physiological gating properties of Ca_*V*_1.3 Ca^2+^-channels (Ortner et al., 2020) at IHC ribbon synapses, RIM-BP1 seems to play a minor – if any – role in regulating the number of synaptic Ca_*V*_1.3 Ca^2+^-channels.

**TABLE 2:** Summary of Ca^2+^-current (I_*Ca*_) data from perforated patch-clamp recordings.

	Amplitude (pA)	V_*half*_ (mV)	Slope factor k
*RIM-BP2^+/+^ (n = 17)*	−157 ± 6	−29.9 ± 0.9	6.9 ± 0.1
*RIM-BP2^–/–^ (n = 16)*	−126 ± 9	−30.0 ± 1.5	6.7 ± 0.2
*RIM-BP1/2^–/–^ (n = 15)*	−131 ± 1	−28.3 ± 0.1	6.9 ± 0.1
*p*-value (*RIM-BP1/2^–/–^ vs. RIM-BP2^+/+^)*	0.02* Wilcoxon rank test	0.3 Student’s *t*-test	0.7 Wilcoxon rank test
*p*-value (*RIM-BP1/2^–/–^ vs. RIM-BP2^–/–^)*	0.7 Student’s *t*-test	0.2 Student’s *t*-test	0.4 Student’s *t*-test

Summary of IHC grand average data from perforated patch-clamp recordings of Ca^2+^-currents (I_*Ca*_) from *RIM-BP1/2^–/–^, RIM-BP2^+/+^* and *RIM-BP2^–/–^* IHCs (Figure 1). Whole-cell I_*Ca*_ was analyzed regarding amplitude, voltage of half-maximal activation (V_*half*_) and voltage-dependence of Ca^2+^-channel gating (slope factor k representing voltage-sensitivity of Ca^2+^-influx). Data represent IHC grand averages, mean ± SEM; *n* = number of IHCs; *p*-values and statistical test are depicted for each dataset, significance level: n.s. *p* ≥ 0.05, *p < 0.05. Data of RIM-BP2-deficient (*RIM-BP2^–/–^*) and control (*RIM-BP2^+/+^*) IHCs were adapted from Krinner et al. (2017).

In order to address the relevance of RIM-BP1 for IHC exocytosis, we measured the exocytic membrane capacitance changes (ΔC_*m*_) in response to voltage-gated Ca^2+^-influx triggered by step depolarizations to -14 mV of varying length (Figure 2A). It is thought that short IHC depolarization durations up to 20 ms (Figure 2C) primarily trigger exocytic release of the RRP (Moser and Beutner, 2000), while longer IHC depolarization durations (>20 ms) (Figure 2B) also probe the sustained phase of SV release that involves SV replenishment to the RRP and subsequent SV fusion (Moser and Beutner, 2000; Schnee et al., 2005; Goutman and Glowatzki, 2007; Meyer et al., 2009; Neef et al., 2009). Sustained exocytosis was not significantly different in *RIM-BP1/2^–/–^* IHCs when compared to control *RIM-BP2^+/+^* IHCs or RIM-BP2-deficient IHCs (Figure 2B, *p*-values are summarized in Table 3). However, RRP exocytosis, probed by short depolarizations (≤20 ms, Figure 2C) was mildly but significantly reduced in RIM-BP1/2-deficient IHCs (*p*_5ms_ = 0.003, *p*_10ms_ = 0.04; one-way ANOVA and *post hoc* Tukey’s test, Table 3), not seen in RIM-BP2-deficient IHCs (Krinner et al., 2017). The effect was largest for 5 ms step depolarizations, where also a significant difference between the RIM-BP1/2-deficient and RIM-BP2-deficient IHCs was detected (*p*_5ms_ = 0.03; one-way ANOVA and *post hoc* Tukey’s test) (Figure 2C and Table 3). The reasons for not finding a significant reduction in sustained exocytosis in *RIM-BP1/2^–/–^* IHCs that we previously found in *RIM-BP2^–/–^* IHCs remain unclear. One might speculate that SVs not released by short depolarizations in *RIM-BP1/2^–/–^* IHCs get recruited later, partially masking the SV replenishment deficit reported for *RIM-BP2^–/–^* IHCs. Moreover, we note that cell-to-cell variability is high for exocytosis in response to longer stimuli, which might also contribute to this discrepancy. In conclusion, our data indicate that both RIM-BPs contribute to SV exocytosis at the IHC ribbon synapse. Among RIM-BPs at the IHC ribbon synapse, RIM-BP1 seems to be required for exocytosis of the RRP, while RIM-BP2 takes a prevailing role in clustering Ca^2+^-channels at the IHC AZ and enabling efficient SV replenishment (Krinner et al., 2017).

**TABLE 3:** Summary of statistical analysis of patch-clamp data.

		5 ms	10 ms	20 ms	50 ms	100 ms	200 ms
ΔC_*m*_	*RIM-BP1/2^–/–^ vs. RIM-BP2^+/+^*	0.003**	0.04*	0.07	0.3	0.3	0.2
	*RIM-BP1/2^–/–^ vs. RIM-BP2^–/–^*	0.03*	0.5	0.8	0.2	0.2	0.3
Q_*real*_	*RIM-BP1/2^–/–^ vs. RIM-BP2^+/+^*	0.6	0.9	0.6	0.3	0.3	0.07
	*RIM-BP1/2^–/–^ vs. RIM-BP2^–/–^*	0.3	0.1	0.1	0.6	0.6	0.9

Summary of statistical analysis of patch-clamp measurement data (Figure 2B): Membrane capacitance changes (ΔC_*m*_, upper columns) and the corresponding whole-cell Ca^2+^-current integrals (Q_*Ca*_, lower columns) during various depolarization durations (5–200 ms) were compared between IHCs of different genotypes: RIM-BP1/2-deficient (*RIM-BP1/2^–/–^*, *n* = 8), RIM-BP2-deficient (*RIM-BP2^–/–^*, *n* = 10) and control (*RIM-BP2^+/+^*, *n* = 12). *p*-values from one-way ANOVA and *post hoc* Tukey’s test; significance level: n.s. *p* ≥ 0.05, *p < 0.05, **p < 0.01. Data of RIM-BP2-deficient (*RIM-BP2^–/–^*) and control (*RIM-BP2^+/+^*) mice were adapted from Krinner et al. (2017).

### Both RIM-BP1 and 2 Are Required for Normal Hearing

**FIGURE 3:**
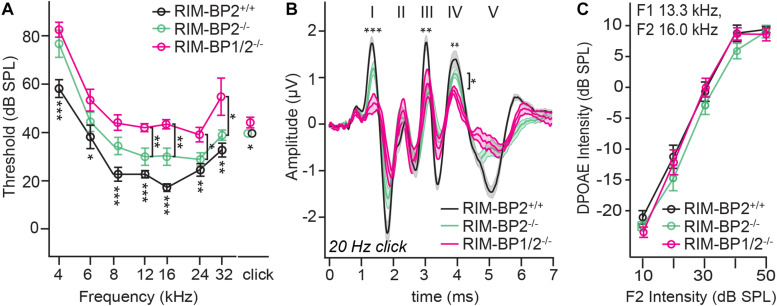
Both RIM-BP1 and -2 are required for normal hearing. **(A)** Compared to control mice (*RIM-BP2^+/+^*, black), auditory brainstem response (ABR) thresholds of RIM-BP1/2-deficient mice (magenta) were elevated for all recorded frequencies (indicated by asterisk on top of traces: 4 kHz: *p* = 0.0007, 6 kHz: *p* = 0.01, 8 kHz: *p* = 0.0001, 12 kHz: *p* < 0.0001, 16 kHz: *p* < 0.0001, 24 kHz: *p* = 0.002, 32 kHz: *p* = 0.003). Additionally, ABR thresholds differed significantly between RIM-BP2-deficient (green) and RIM-BP1/2-deficient mice at frequencies ≥12kHz (indicated by asterisk and bracket: 12 kHz: *p* = 0.003, 16 kHz: *p* = 0.004, 24 kHz: *p* = 0.03, 32 kHz: *p* = 0.02). Click thresholds of RIM-BP1/2-deficient mice showed significant difference to both, control as well as RIM-BP2-deficient mice (*p* = 0.01 and *p* = 0.03, respectively). **(B)** ABR waves represent the compound action potential of different neuronal populations along the auditory pathway during 20 Hz stimulation. Compared to control mice (black), ABR waveforms (80 dB peak equivalent, 20 Hz stimulation rate) of RIM-BP1/2-deficient mice (magenta) showed significantly smaller amplitudes (indicated by asterisk on top of traces: wave I: *p* = 0.0006, wave III: *p* = 0.001, wave IV: *p* = 0.005). In addition, ABR wave IV amplitude differed significantly between RIM-BP2-deficient (green) and RIM-BP1/2-deficient mice (indicated by asterisk and bracket: *p* = 0.03). **(C)** Mechanoelectrical transduction and cochlear amplification at the frequency of strongest hearing threshold increase [16 kHz, see panel **(A)**] were assessed through recordings of otoacoustic emissions (DPOAE) (F1: 13.3 kHz, F2: 16 kHz). DPOAE amplitudes were unaltered in RIM-BP1/2-deficient mice compared to control mice, suggesting normal function upstream of synaptic sound encoding at IHC synapses. **(A–C)** *RIM-BP1/2^–/–^* (*n* = 7), *RIM-BP2^+/+^* (*n* = 11), *RIM-BP2^–/–^* (*n* = 8); Data information: Data represent grand averages, mean ± SEM; one-way ANOVA and *post hoc* Tukey’s multiple comparison test, *p*-values are summarized in Table 4. Significance level: n.s. *p* ≥ 0.05, *p < 0.05, **p < 0.01, ***p < 0.001; *n* = number of mice; age of mice: 8–10 weeks. Data of RIM-BP2-deficient (*RIM-BP2^–/–^*) and control (*RIM-BP2^+/+^*) mice were adapted from Krinner et al. (2017).

Finally, we investigated whether additional genetic disruption of RIM-BP1 would cause a synaptopathic hearing impairment exceeding that found in mice lacking RIM-BP2 alone (Krinner et al., 2017). For that, we recorded ABRs from *RIM-BP1/2^–/–^* mice. ABR waves reflect the compound neural action potential firing along the auditory pathway initiated at the first auditory synapse between sensory IHCs and SGNs. Hearing thresholds (Figure 3A) were determined as the lowest stimulus intensity that evoked a reproducible response ABR waveform. Compared to the hearing thresholds of *RIM-BP2^+/+^* control mice, we found a significant threshold elevation by on average 20 dB SPL (SD ± 4.0 dB SPL) for all recorded frequencies in *RIM-BP1/2^–/–^* mice (*p*_4kHz_ = 0.0007, *p*_6kHz_ = 0.01, *p*_8kHz_ = 0.0001, *p*_12kHz_ < 0.0001, *p*_16kHz_ < 0.0001, *p*_24kHz_ = 0.002, *p*_32kHz_ = 0.003; one-way ANOVA and *post hoc* Tukey’s multiple comparison test). Interestingly, there was a significant threshold elevation also compared to *RIM-BP2^–/–^* mice (*p*_12kHz_ = 0.003, *p*_16kHz_ = 0.004, *p*_24kHz_ = 0.03, *p*_32kHz_ = 0.02; one-way ANOVA and *post-hoc* Tukey’s multiple comparison test). Along the same lines, the ABR wave amplitudes (Figure 3B and Table 4) were strongly reduced in *RIM-BP1/2^–/–^* mice compared to *RIM-BP2^+/+^* during 20 Hz click stimulation (wave I: *p* = 0.0006, wave III: *p* = 0.001, wave IV: *p* = 0.005; one-way ANOVA and *post-hoc* Tukey’s multiple comparison test). When we compared the ABRs of *RIM-BP1/2^–/–^* mice to *RIM-BP2^–/–^* mice at 20 Hz click stimulation, we found a significant ABR wave amplitude decrease in wave IV (*p* = 0.03; one-way ANOVA and *post hoc* Tukey’s multiple comparison test), which likely reflects additional deficits in synaptic transmission in the auditory brainstem. In addition, the ABR wave I amplitude difference between *RIM-BP1/2^–/–^* mice and *RIM-BP2^–/–^* mice reached statistical significance when increasing the rate click stimulation to 100 Hz (*p* = 0.03; one-way ANOVA and *post hoc* Tukey’s multiple comparison test; Table 4). Since ABR wave I reflects synchronized firing of the SGNs induced by synaptic transmission from sensory IHCs, these data suggest a role of RIM-BP1 either in the sensory IHCs themselves or in the SGNs, downstream of the IHC ribbon synapse. To exclude that the hearing threshold elevation of RIM-BP1/2-deficient mice is caused by impaired outer hair cell-mediated cochlear amplification, we also recorded DPOAEs (Figure 3C). We found normal DPOAE amplitudes in *RIM-BP1/2^–/–^* mice (for all F2 intensities: *p* > 0.05; one-way ANOVA and *post hoc* Tukey’s multiple comparison test), indicating preserved outer hair cell function, which is keeping with our previous findings in RIM-BP2-deficient mice (Krinner et al., 2017).

**TABLE 4:** Summary of statistical analysis of ABR data.

		Wave I amplitude	Wave II amplitude	Wave III amplitude	Wave IV amplitude	Wave V amplitude
20 Hz	*RIM-BP1/2^–/–^ vs. RIM-BP2^+/+^*	0.0006***	0.9	0.001**	0.005**	0.5
	*RIM-BP1/2^–/–^ vs. RIM-BP2^–/–^*	0.2	0.9	0.6	0.03*	0.8
100 Hz	*RIM-BP1/2^–/–^ vs. RIM-BP2^+/+^*	0.0004***	0.8	0.002**	0.002**	0.5
	*RIM-BP1/2^–/–^ vs. RIM-BP2^–/–^*	0.03*	0.8	0.008**	0.05	0.7

Summary of statistical analysis of ABRs (Figure 3B): Wave I-V amplitudes were compared between *RIM-BP1/2^–/–^* (*n* = 7), *RIM-BP2^+/+^* (*n* = 11) and *RIM-BP2^–/–^* (*n* = 8) mice for 20 or 100 Hz click stimulation. *p*-values from one-way ANOVA and *post hoc* Tukey’s test; significance level: n.s. *p* ≥ 0.05, *p < 0.05, **p < 0.01, ***p < 0.001. Data of RIM-BP2-deficient (*RIM-BP2^–/–^*) and control (*RIM-BP2^+/+^*) mice were adapted from Krinner et al. (2017).

## Discussion

Here we probed for a role of RIM-BP1 in synaptic sound encoding at the IHC-SGN synapse. Building on our prior analysis of mice lacking RIM-BP2 (Krinner et al., 2017), we now studied the effects of additional deletion of RIM-BP1. *RIM-BP1/2^–/–^* mice showed an aggravation of the auditory synaptopathy (Moser and Starr, 2016) phenotype, with further elevation of the hearing threshold and stronger reduction of ABR wave amplitudes than found in *RIM-BP2^–/–^* mice, despite normal cochlear amplification. Our recent work suggests that, indeed, RIM-BP1 and 2 both play a role in mammalian hearing with a leading role of RIM-BP2 and partial functional redundancy of RIM-BP1 and 2. Scrutinizing the requirement for the presence of both RIM-BPs indicated a role for high frequency synaptic transmission *in vivo*, where the effect of additional RIM-BP1 deletion on ABR thresholds and amplitudes was enhanced at higher stimulation frequencies.

Mechanistically, such a hearing impairment could be caused by either a deficit in presynaptic transmitter release from sensory IHCs or a functional defect of the postsynaptic SGNs. On the presynaptic side, a reduction of IHC transmitter release could be caused indirectly via impaired Ca^2+^-influx and/or directly via immediate effects on SV release. We found that at IHC ribbon synapses, additional deletion of RIM-BP1 did not further alter voltage-gated Ca^2+^-influx beyond the reduction found in RIM-BP2 deficient IHCs. In contrast to our data on IHCs, combined genetic deletion of RIM-BP1 and -2 in rod bipolar cell AZs, reduced the number of Ca^2+^-channels more strongly than the deletion of the individual RIM-BPs (Luo et al., 2017). The same study also showed a significant compensatory elevation of RIM protein levels in RIM-BP1/2-deficient retinae (Luo et al., 2017). Other studies pointed toward redundant functions of RIMs and RIM-BPs in promoting the synaptic abundance of Ca^2+^-channels: The combined deletion of RIMs and RIM-BPs reduced Ca^2+^-channel abundance (Kushibiki et al., 2019) or Ca^2+^-triggered release (Acuna et al., 2016) more drastically than would have been expected from the effects observed upon the deletion of individual genes. Since the impact of RIMs (Jung et al., 2015) and RIM-BP2 (Krinner et al., 2017) on synaptic Ca_*V*_1.3 Ca^2+^-channel abundance in IHCs has been shown, one could argue, that either RIM-BP1 has only a minor role on synaptic Ca^2+^-channel abundance or that its effect was masked by an increased abundance of other AZ proteins like RIMs due to compensatory upregulation upon the combined loss of both RIM-BP1 and 2. Such compensatory upregulation of other multidomain proteins of the AZ should be tested in future studies that might also test the possibility of an upregulated expression of RIM-BP1 in RIM-BP2-deficient IHCs.

Then, the aggravation of the auditory synaptopathy observed in *RIM-BP1/2^–/–^* mice might point to a direct involvement of RIM-BP1 in SV exocytosis. Indeed, upon RIM-BP1/2 deletion, we found a subtle but significant reduction of synchronous SV exocytosis triggered by brief step-depolarizations not found in *RIM-BP2^–/–^* mice (Figure 2B) likely resulting in less synchronized SGN activation and, hence, smaller ABR wave I amplitude in *RIM-BP1/2^–/–^* mice (Figure 3B). A correspondence of impaired RRP exocytosis and reduced wave amplitude has previously been reported for bassoon-deficient IHCs (Khimich et al., 2005; Buran et al., 2010). However even though exocytosis is nearly completely abolished during *in vitro* capacitance measurements with 5 ms step depolarization, the ABR wave I recorded *in vivo* did not show such a dramatic amplitude reduction. We consider several possible and non-exclusive explanations for the discrepancy of the *in vivo* and *in vitro* data. (i) There is greater experimental variability at the level of *in vitro* patch-clamp recordings from individual IHCs containing just a dozen of synapses with stimulation repeated only 2–3 times per IHC, while ABR in response to 80 dB click reflect sound encoding of thousands of IHC-SGN synapse averaged over 1,000 trials. (ii) The age differs between the recordings of the *in vitro* patch-clamp C_*m*_ measurements (2–3 week-old mice) and the *in vivo* ABR measurements (8–10 week-old mice) which could influence synaptic transmission due to synapse maturation beyond 2–3 weeks. Morphological studies pointed out that cochlear thresholds and the subdivisions of SGNs according to their spontaneous firing rate are mature only after 28 days, which is especially critical for *in vivo* studies (Liberman and Liberman, 2016). (iii) The *in vitro* patch-clamp C_*m*_ measurements were done at room temperature, whereas ABR recordings were done at 37°C (mammalian body temperature). *In vitro* experiments done in frog and mammalian auditory hair cells showed, that at higher temperatures, the Ca^2+^-current activation kinetics were accelerated and amplitudes increased, which reduces synaptic delay of glutamate release and supports synchronization of SV release (Nouvian, 2007; Chen and von Gersdorff, 2019). In addition, the authors found a temperature-dependent increase in synchronous exocytosis, which was not only due to the accelerated Ca^2+^-current, but likely due to a temperature-dependent increase in efficiency of Ca^2+^ influx triggered SV exocytosis (Nouvian, 2007). These differences could explain the discrepancy between the strongly reduced response to 5 ms depolarization and the lesser reduction of ABR wave I amplitude. Thus, it would be interesting to perform the patch-clamp recordings at physiological temperature. (iv) The peak preceding wave I in Figure 3B is likely the summating potential (SP), thought to reflect the synchronous depolarization of IHCs (Whitfield and Ross, 1965; Dallos et al., 1972). That the SP is unaffected by loss of RIM-BP1 and/or RIM-BP2 indicated that the mechanotransduction machinery is unaffected, supporting a conclusion that the reduction in wave I amplitude is not due to a deficit upstream of the ribbon synapse, but rather reflects reduced activation of SGNs or desynchronization of SGN activation. Indeed, the link of RRP exocytosis in IHCs to synchronous SGN activation and ABR wave I amplitude has been established (Khimich et al., 2005; Wittig and Parsons, 2008; Buran et al., 2010; Rutherford et al., 2012; Li et al., 2014).

As for other synapses, RIM-BPs might be involved in a tight Ca^2+^-channel-SV coupling (Liu et al., 2011; Acuna et al., 2015; Grauel et al., 2016; Luo et al., 2017). While tight Ca^2+^-channel-SV coupling seems preserved in *RIM-BP2^–/–^* IHCs at least after RRP recovery from depletion (Krinner et al., 2017), deletion of both RIM-BP1 and 2 might impair this coupling and, consequently, synchronous SV release (Figure 2C). Alternatively, subpools of the RRP might exist (Goutman and Glowatzki, 2007) and be differently dependent on RIM-BP1 and 2. Future experiments, e.g., using paired recordings (Goutman and Glowatzki, 2007) or dual color imaging of presynaptic Ca^2+^ signals and glutamate release (Özçete and Moser, 2020) should address these possibilities for individual IHC synapses. The better preserved sustained exocytosis in *RIM-BP1/2^–/–^* double-knockout IHCs compared to *RIM-BP2^–/–^* single knockout IHCs might then simply reflect a partial masking of impaired SV replenishment (Krinner et al., 2017) by the protracted release of the RRP in the absence of both RIM-BPs.

Another remaining task for future experiments is to further scrutinize RIM-BP1 expression in the organ of Corti. As described the RIM-BP1 antibodies at our disposal did not provide sufficient specificity to address this point. Interestingly, a recent transcript analysis of mouse IHCs reported expression of RIM-BP2 and -3, but not RIM-BP1 in IHCs (Ortner et al., 2020). This is consistent with our findings from two different approaches of expression analysis where we failed to detect RIM-BP1 mRNA in IHCs by single-cell RT-PCR and the RNAscope data did not strongly support RIM-BP1 mRNA expression in IHCs. However, our RT-PCR data showed a clear mRNA expression of RIM-BP1 in the organ of Corti. Future experiments should revisit a putative RIM-BP1 expression in IHCs e.g., by using RIM-BP1 specific immunolabeling or RNA sequencing of IHCs and also address a potential upregulated expression of RIM-BP1 in RIM-BP2-deficient IHCs. Moreover, alternative mechanisms explaining the aggravated hearing impairment found in RIM-BP1/2-deficient mice should be addressed which could involve a potential functional RIM-BP1 expression in SGNs or efferent olivocochlear neurons. If expressed in SGNs, it would be interesting to check for a putative postsynaptic function of RIM-BP1, e.g., by interacting and regulating postsynaptic receptor- or ion channel properties (Nirenberg and Yifrach, 2020). More speculatively, one could consider of a transsynaptic regulation from SGNs towards IHC AZs, leading to an impairment in synchronous exocytosis of the RRP in IHCs due to the loss of RIM-BP1. Such transsynaptic coupling of IHCs and SGNs was described for the AMPA receptor – PSD-95 complex, which is required for the correct spatial alignment of Ca_*V*_1.3 Ca^2+^-channels across the synaptic cleft (Fell et al., 2016).

## Author’s Note

RIM-binding proteins are multidomain proteins of the presynaptic active zone that interact with Ca^2+^ channels and other proteins forming release sites for synaptic vesicles. At the inner hair cell (IHC) ribbon synapse, RIM-BP2 tethers voltage-gated Ca^2+^-channels and promotes sustained exocytosis. In the present study, we probed for a function of RIM-BP1, another RIM-BP family member. We found that disruption of RIM-BPs 1 and 2 in mice causes a synaptopathic hearing impairment exceeding that of mice lacking only RIM-BP2. While the reduction of Ca^2+^-influx seemed comparable between IHCs of both mutant genotypes, deletion of both RIM-BPs caused an additional impairment of synchronous exocytosis of the readily releasable pool of synaptic vesicles. Hence, while RIM-BP2 appears to be the dominant isoform, both RIM-BPs are required for normal sound encoding at the inner hair cell ribbon synapse.

## Data Availability Statement

The original contributions presented in the study are included in the article/Supplementary Material, further inquiries can be directed to the corresponding author.

## Ethics Statement

The animal study was reviewed and approved by University of Göttingen Board for Animal Welfare and Animal Welfare Office of the State of Lower Saxony. Written informed consent was obtained from the owners for the participation of their animals in this study.

## Author Contributions

SK and TM designed the study. SK performed immunohistochemistry, RNAscope and confocal microscopy, and patch-clamp capacitance measurements. FP performed RT-PCR experiments. DB and CV contributed to the RNAscope experiments. TM and SK prepared the manuscript with contributions of all authors.

## Conflict of Interest

The authors declare that the research was conducted in the absence of any commercial or financial relationships that could be construed as a potential conflict of interest.

## Supplementary Material

Supplementary Figure 1 Efforts to establish immunohistochemistry for RIM-BP1 in the mouse cochlea. Maximum projections of confocal stacks after immunohistochemistry from apical organ of Corti whole-mount explants from 2-week-old *C57BL/6* and *RIM-BP1/2^–/–^* mice. RIM-BP1 immunofluorescence (green) co-localizes with presynaptic ribbons (CtBP2/RIBEYE, magenta) and either **(A)** Bassoon (blue) or **(B)** Synapsin1/2 (blue) in *C57BL/6* IHCs. Synapsin1/2 marks presynaptic terminals of efferent lateral olivocochlear neurons projecting onto type I SGN synapses. RIM-BP1 is not absent in parallel processed *RIM-BP1/2*^–/–^ IHCs, questioning the RIM-BP1 antibody specificity. Scale bar: 5 μm.

Supplementary Figure 2 RNA Analysis of RIM-BP1 expression in the organ of Corti. Maximum projection of confocal stack after RNAscope experiment and consecutive immunohistochemistry from apical organ of Corti whole-mount explant from 2-week-old *C57BL/6* mouse. Calretinin [**(B)**, blue in merge image **(A)**] marks IHCs. Otoferlin RNAscope probe [**(C)**, magenta in merge image **(A)**] was used as positive control. Otoferlin mRNA is present in IHCs, mainly localized around the IHC nuclei. RIM-BP1 mRNA could not be detected by the RNAscope probe [**(D)**, green in merge image **(A)**]. Scale bar: 10 μm.

Supplementary Figure 3 RIM-BP1 is expressed in the mouse organ of Corti. Nested RT-PCR from organs of Corti, brain, and 10 individual IHCs from 2-week-old *C57Bl/6* mice. In the negative control cDNA was omitted from the reaction. Nested PCR primers were specific for RIM-BP1 **(A)** or RIM-BP2 **(B)** cDNA. The following DNA bands were expected: 196 bp for RIM-BP1 **(A)**, 408 bp for RIM-BP2 **(B)**. Both RIM-BPs were detected in brain tissue. RIM-BP2 mRNA was detected in individual IHCs. RIM-BP1 was detected in the organ of Corti, but not in individual IHCs.
