# Supplementary figures and images for: RIM-Binding Proteins Are Required for Normal Sound-Encoding at Afferent Inner Hair Cell Synapses
Source: Front Mol Neurosci. 2021 Mar 23;14:651935. doi: 10.3389/fnmol.2021.651935 (PMC8044855; doi:10.3389/fnmol.2021.651935)

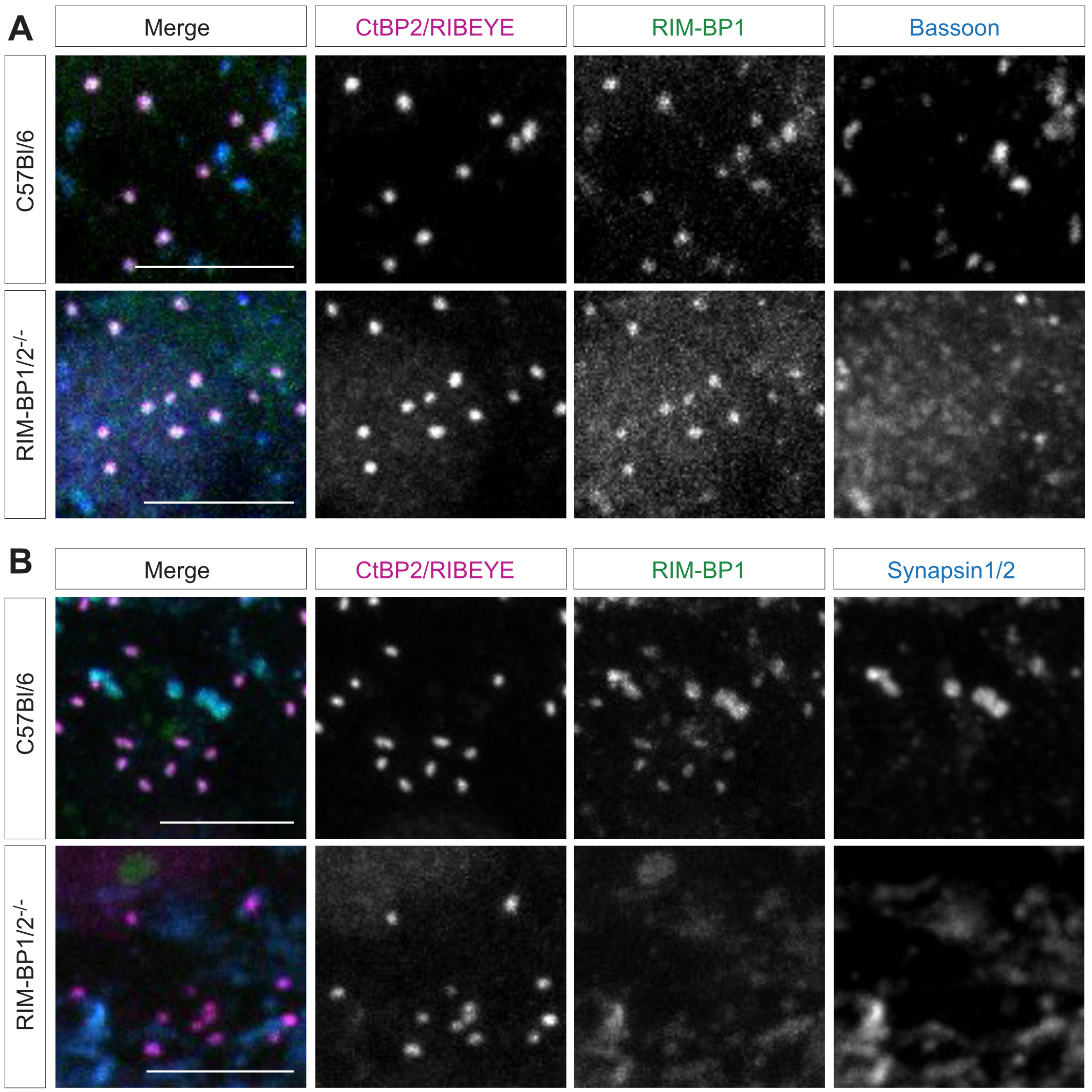

Supplement: Supplementary Figure 1 — Efforts to establish immunohistochemistry for RIM-BP1 in the mouse cochlea. Maximum projections of confocal stacks after immunohistochemistry from apical organ of Corti whole-mount explants from 2-week-old C57BL/6 and RIM-BP1/2–/– mice. RIM-BP1 immunofluorescence (green) co-localizes with presynaptic ribbons (CtBP2/RIBEYE, magenta) and either (A) Bassoon (blue) or (B) Synapsin1/2 (blue) in C57BL/6 IHCs. Synapsin1/2 marks presynaptic terminals of efferent lateral olivocochlear neurons projecting onto type I SGN synapses. RIM-BP1 is not absent in parallel processed RIM-BP1/2–/– IHCs, questioning the RIM-BP1 antibody specificity. Scale bar: 5 μm. [file Image_1.tiff]

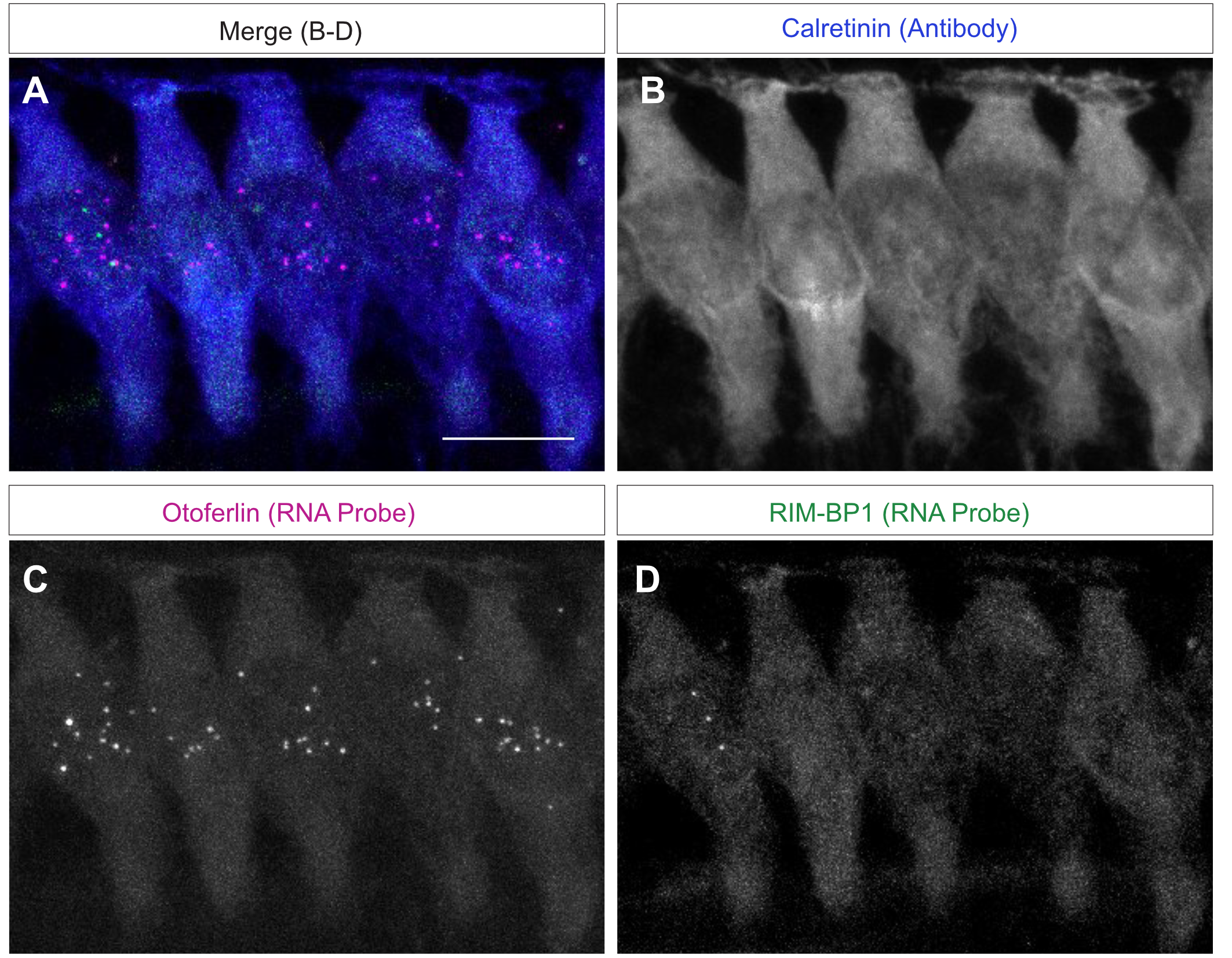

Supplement: Supplementary Figure 2 — RNA Analysis of RIM-BP1 expression in the organ of Corti. Maximum projection of confocal stack after RNAscope experiment and consecutive immunohistochemistry from apical organ of Corti whole-mount explant from 2-week-old C57BL/6 mouse. Calretinin [(B), blue in merge image (A)] marks IHCs. Otoferlin RNAscope probe [(C), magenta in merge image (A)] was used as positive control. Otoferlin mRNA is present in IHCs, mainly localized around the IHC nuclei. RIM-BP1 mRNA could not be detected by the RNAscope probe [(D), green in merge image (A)]. Scale bar: 10 μm. [file Image_2.tiff]

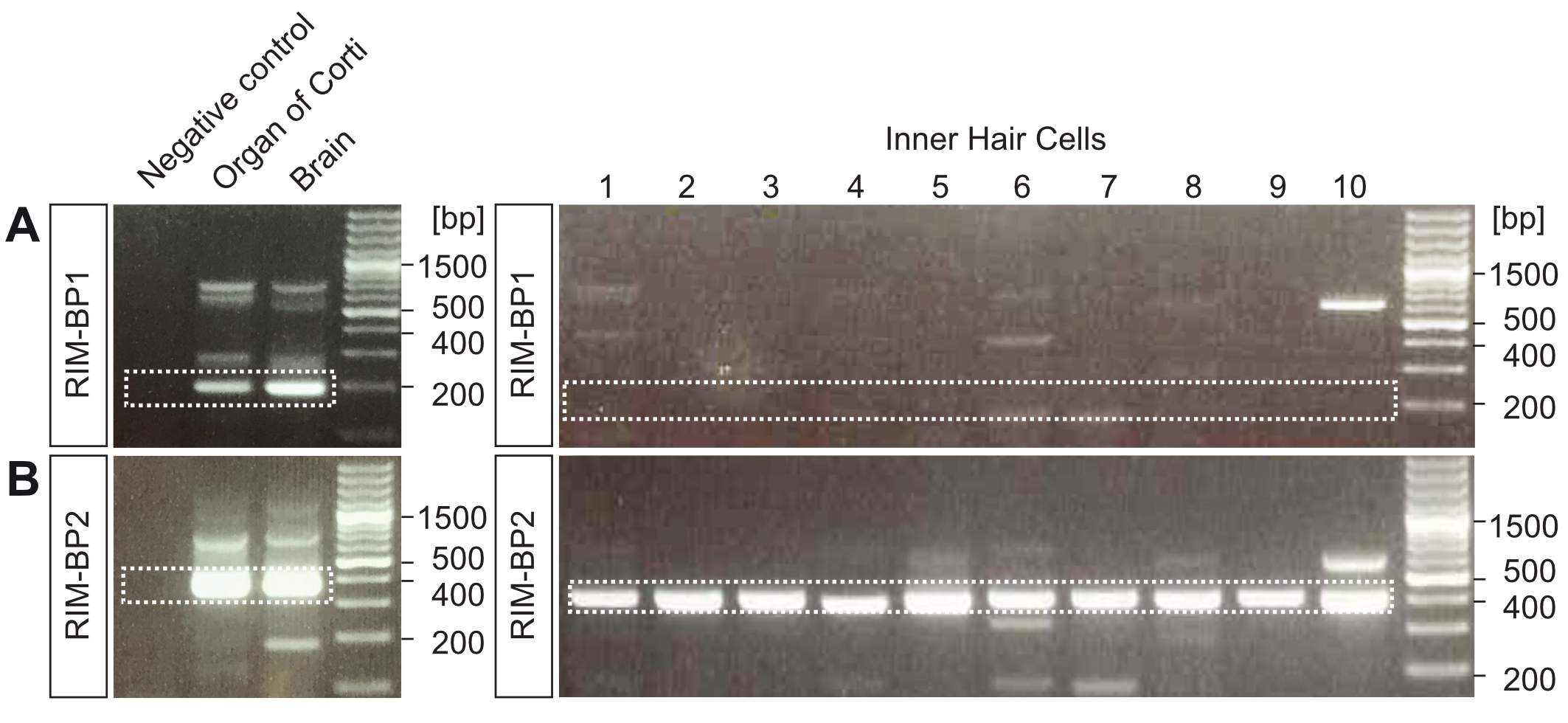

Supplement: Supplementary Figure 3 — RIM-BP1 is expressed in the mouse organ of Corti. Nested RT-PCR from organs of Corti, brain, and 10 individual IHCs from 2-week-old C57Bl/6 mice. In the negative control cDNA was omitted from the reaction. Nested PCR primers were specific for RIM-BP1 (A) or RIM-BP2 (B) cDNA. The following DNA bands were expected: 196 bp for RIM-BP1 (A), 408 bp for RIM-BP2 (B). Both RIM-BPs were detected in brain tissue. RIM-BP2 mRNA was detected in individual IHCs. RIM-BP1 was detected in the organ of Corti, but not in individual IHCs. [file Image_3.tiff]
